# Does hospital volume affect outcomes after abdominal cancer surgery: an analysis of Swiss health insurance claims data

**DOI:** 10.1186/s12913-022-07513-5

**Published:** 2022-02-26

**Authors:** Kevin Wirth, Markus Näpflin, Sereina M. Graber, Eva Blozik

**Affiliations:** 1 Helsana Group, Department of Health Sciences, Zurich, Switzerland; 2 University of Lucerne, Department of Health Sciences and Medicine, Lucerne, Switzerland; 3 University of Zurich, Institute of Primary Care, Zürich, Switzerland

**Keywords:** Hospital volume, Volume-outcome, Abdominal cancer, Treatment concentration, Hospital planning

## Abstract

**Background:**

Medical treatment quality has been shown to be better in high volume than in low volume hospitals. However, this relationship has not yet been confirmed in abdominal cancer in Switzerland and is relevant for referral of patients and healthcare planning. Thus, the present study investigates the association between hospital volumes for surgical resections of colon, gastric, rectal, and pancreatic carcinomas and outcomes.

**Methods:**

This retrospective analysis is based on anonymized claims data of patients with mandatory health insurance at Helsana Group, a leading health insurance in Switzerland. Outcome parameters were length of hospital stay, mortality and cost during the inpatient stay as well as at 1-year follow-up. Hospital volume information was derived from the Quality Indicators dataset provided by the Swiss Federal Office of Public Health. The impact of hospital volume on the different treatment outcomes was statistically tested using generalized estimating equations (GEE) models, taking into account the non-independence of observations from the same hospital.

**Results:**

The studies included 2′859 resections in patients aged 18 years and older who were hospitalized for abdominal cancer surgery between 2014 and 2018. Colon resections were the most common procedures (*n* = 1′690), followed by rectal resections (*n* = 709). For rectal, colon and pancreatic resections, an increase in the mean number of interventions per hospital and a reduction of low volume hospitals could be observed. For the relationship between hospital volume and outcomes, we did not observe a clear dose-response relationship, as no significantly better outcomes were observed in the higher-volume category than in the lower-volume category. Even though a positive “routine effect” cannot be excluded, our results suggest that even hospitals with low volumes are able to achieve comparable treatment outcomes to larger hospitals.

**Conclusion:**

In summary, this study increases transparency on the relationship between hospital volume and treatment success. It shows that simple measures such as defining a minimum number of procedures only might not lead to the intended effects if other factors such as infrastructure, the operating team or aggregation level of the available data are not taken into account.

**Supplementary Information:**

The online version contains supplementary material available at 10.1186/s12913-022-07513-5.

## Background

Providing high quality treatment is a key goal in cancer care and is characterized by complex interaction of clinical judgment, surgical skills and multidisciplinary care [1, 2]. Over the last decades, quality of oncological care was enhanced by progresses in the understanding of cancer biology, pathology, radiation oncology, interventional radiology and systemic therapy in combination with the rapid advances of molecular analysis and imaging techniques [3, 4].

Numerous international studies have examined the volume-quality relationship for high-risk abdominal cancer surgery [5–23] and the large majority showed clear, beneficial effects of increasing volume on outcomes (primarily mortality and length of stay). At the structural level, high volume hospitals are assumed to contribute to better patient outcomes by offering expertise through multidisciplinary teamwork and enhanced implementation of evidence-based guidelines [24–26]. At the individual level, a surgical team is able to minimize blood loss and operative time as well as intra- and postoperative complications by gaining experience [9, 20, 27–29]. Studies indicating a positive relationship between hospital volume and treatment quality provide the scientific fundament for minimum case numbers introduced as part of quality control in inpatient care in Switzerland [30–32]. Hence, hospitals must demonstrate a defined number of operations in order to be awarded service contracts. However, the applicability of previous studies on volume and outcome to current practice is controversial. First, many studies on volume and outcome are outdated. Given that surgical mortality for many procedures has decreased substantially since these studies were conducted [33–38], the relative impact of the volume effect of procedures may have decreased. Second, studies showing strong association between hospital volumes and better outcomes have been predominantly conducted in larger countries (i.e., US or UK). However, these results are not easily transferrable to healthcare systems with relatively small geographic units, small patient numbers and / or a small-spatial dispersion of hospitals. Consequently, the focus of our work was 1) to describe distribution and temporal changes in hospital volumes regarding colon, gastric, rectal and pancreatic resections between 2014 and 2018 in Switzerland and 2) to investigate whether high volume hospitals perform better with respect to postoperative mortality, length of hospital stay and cost.

## Methods

### Study design and study population

This retrospective analysis is based on anonymized claims data of patients with mandatory health insurance at Helsana Group, a leading health insurance in Switzerland. Hospital volume information was added based on data from the Quality Indicators dataset publicly available from the Federal Office of Public Health (FOPH) [39]. The Helsana Group covers a market share of 16% across all geographic regions, so that these data are assumed to be largely representative for the general population of Switzerland [40, 41]. The basic health service package provided by the basic mandatory health insurance system is administered federally and includes a broad catalog of diagnostic, therapeutic and rehabilitative services in the ambulatory and inpatient setting with good access to healthcare for Swiss residents [42].

The study population included 2′859 resections in patients aged 18 years and older who were hospitalized for abdominal cancer surgery between 2014 and 2018 and who had mandatory health insurance at Helsana Insurance Group. Patients with multiple resections were excluded from the sample.

### Definitions

The Inpatient Quality Indicators (IQI) specifications from Swiss acute care hospitals routinely collected by the Swiss FOPH are publicly available [39] and were used for definition of abdominal cancer types and for classification of the corresponding volumes per hospital. As IQI specifications are continuously developed, a version is typically valid for 2 years. Thus, IQI version 4.0 was used for the time period 2013–2014, IQI version 4.2 for 2015–2016 and IQI version 5.1 for 2017–2018. The following codes were used: Colon resections / colorectal cancer E4.2 and E4.3, rectal resections / colorectal carcinoma E4.4, gastric resections without esophageal intervention / gastric carcinoma E5.1 and pancreatic resections for malignant neoplasms of the pancreas (age > 19) E7.2. Non-cancer-specific codes for abdominal resections were not considered (E4.1, E5.3, E6.1). The corresponding total annual numbers of interventions at hospital level for each cancer type were retrieved from the federal Quality Indicators dataset. Only those hospitals with at least one treatment per observation year were considered. For tumor resection procedures, the observation period 2014–2018 was examined, except for pancreas resections for which specifications are only available from 2015 onwards as they have been defined for the first time with version 4.2. Based on this codification, we examined the temporal development of hospital volumes for concentration trends. Concentration of procedures are understood as increase in the number of annual resections and the simultaneous reduction of hospitals with very low volumes over a defined period of time [43, 44]. Furthermore, in order to facilitate the description and interpretation of the results, the terms “low” and “high” hospital volumes are used below to denote the category with the highest and lowest case numbers, respectively. The “intermediate” group encompasses all hospitals whose volume ranges between the upper limit of the lowest volume category and the lower limit of the highest volume category.

Outcomes on patient level were all obtained from the health insurance claims database. Length of hospital stay was defined as the time span between the hospital admission date and the discharge date. Patient clinical complexity level (PCCL) is based on Diagnosis Related Groups (DRGs) classification codes and represents the patient-related severity of a given inpatient procedure. The PCCL measure is a multilevel indicator, ranging from severity level 0 (no complication or comorbidity) to 4 (extremely severe complication or comorbidity). General comorbidity of a patient prior to hospital admission was assessed using the number of Pharmaceutical Cost Groups (PCG) per patient [45]. PCGs are an established proxy for presence of chronic diseases using information on reimbursed medications. Further patient characteristics included information about sex, age class, type of health insurance plan and place of residence. Postoperative cost and mortality rates were assessed during twelve months after hospital discharge (follow-up). In Switzerland, basic health insurance is mandatory, and all Swiss residents need to choose their personal health insurance provider and insurance plan. Insurance plans vary by the chosen annual deductible (having a low deductible = CHF 300, 500, yes/no) and selection of a managed care plan (managed care model, yes/no). Patients’ residence was described by considering the type of region (urban vs. rural) and the three major language regions of Switzerland (German, French, Italian). These areas were classified based on homogeneity in the geographical and environmental characteristics, as well as population profiles from the Swiss Federal Office of Statistics (FOS) [46].

### Statistical analysis

To analyze the study populations of the four included cancer entities, descriptive statistics were used. When examining the change in service provision over time and the association between hospital volume and outcomes, hospitals were divided into approximately equal-sized categories (colon resection: ≤20, 21–50, 51–80, > 80; rectal resection: ≤10, 11–20, 21–30, > 30; pancreatic resection: ≤15, 16–30, 31–40, > 40) to balance statistical power across categories. Because of the low volume of gastric resections, hospitals were divided into two hospital volume categories (≤10, > 10).

The effects of hospital volumes on the various treatment outcomes were tested using generalized estimating equations (GEE) (with logit link for binary response variables and gaussian link for continuous response variables), taking into account the clustering of patients within hospitals. Separate GEE models were calculated for each cancer type, using data from the entire observation period. The models additionally control for the following potentially confounding variables: age, sex, language region, comorbidity, and patient-specific overall severity. Other possible covariates, including deductible, insurance model and type of region, were excluded from the models because they did not show any effect on the response variables nor affected the main effects of interest. Furthermore, the interaction between PCCL and volume was examined to test for a potential dependence of the volume effect on severity during surgery. The interaction effects showed no statistical significance and were therefore excluded from the models. For the binomial models, odds ratios (OR) and corresponding confidence intervals were calculated based on the effect sizes. To achieve uniformly distributed residuals around zero for the gaussian models and thus meet the model assumptions, all continuous variables had to be box-cox transformed. The Box-Cox transformation is given by $\mathrm{f}\ \left(\mathrm{x};\uplambda \right)=\frac{{\mathrm{x}}^{\uplambda}-1}{\uplambda}$, if λ not 0, log(x) otherwise. For each model the optimal lambda (λ) was determined based on the maximum log-likelihood. For inpatient cost in the 12-months follow-up period two separate models were calculated: (1) binomial model with inpatient cost > 0 vs. 0 including all patients, (2) gaussian model with box-cox transformed dependent variable for inpatient cost > 0. In order to rule out precision/power loss due to categorization of the volume variable, all models were rerun using the continuous predictor variable. The corresponding results remain unchanged and are available upon request. The models are documented in detail in Tables A1-A4 in the Additional file 1: Appendix.

## Results

Throughout the entire observation period, colon resections were the most common interventions (*n* = 1′690, 59%), whereas gastric resections were the least frequent procedures (*n* = 188, 7%). Over the observation period, the yearly average number of performing hospitals was highest for colon resections (*n* = 93), followed by hospitals for rectal resections (*n* = 78) and gastric resections (*n* = 62), with the lowest average number of performing hospitals for pancreatic resections (*n* = 35). Of all resections, 4% lead to death in the hospital, another 12% within twelve months after hospital discharge and 84% were not fatal after 12 months. Of the 2′859 abdominal resections, 47% were performed on women. The mean age of operated patients was 71 years in men and to 72 years in women. Table 1 depicts the patient characteristics of the study population.

**Table 1:** Sociodemographic characteristics of the study population

Characteristics	Overall, N = 2′859^*a*^	Colon resection *N* = 1′690^*a*^	Rectal resections *N* = 709^*a*^	Gastric resections, *N* = 188^*a*^	Pancreatic resections *N* = 272^*a*^
*Sex*					
Male	1′504 (53%)	842 (50%)	423 (60%)	105 (56%)	134 (49%)
Female	1′355 (47%)	848 (50%)	286 (40%)	83 (44%)	138 (51%)
*Age group*					
20–65	752 (26%)	354 (21%)	235 (33%)	68 (36%)	95 (35%)
66–70	445 (16%)	250 (15%)	115 (16%)	23 (12%)	57 (21%)
71–75	470 (16%)	271 (16%)	113 (16%)	40 (21%)	46 (17%)
76–80	496 (17%)	310 (18%)	111 (16%)	29 (15%)	46 (17%)
81–85	398 (14%)	278 (16%)	74 (10%)	21 (11%)	25 (9%)
86–90	236 (8%)	177 (10%)	51 (7%)	5 (3%)	3 (1%)
≥ 91	62 (2%)	50 (3%)	10 (1%)	2 (1%)	0 (0%)
*Type of residence*					
Agglomeration	515 (18%)	304 (18%)	31 (16%)	60 (22%)	120 (17%)
Rural	399 (14%)	230 (14%)	27 (14%)	40 (15%)	102 (14%)
Urban	1′945 (68%)	1′156 (68%)	130 (69%)	172 (63%)	487 (69%)
*Language region*					
German	2′183 (76%)	1′249 (74%)	134 (71%)	221 (81%)	579 (82%)
Italian	355 (12%)	237 (14%)	23 (12%)	24 (9%)	71 (10%)
French	321 (11%)	204 (12%)	31 (16%)	27 (10%)	59 (8%)
*Deductible*					
Low	2′430 (85%)	1′459 (86%)	159 (85%)	224 (82%)	588 (83%)
High	429 (15%)	231 (14%)	29 (15%)	48 (18%)	121 (17%)
*Insurance model*					
Standard	1′406 (49%)	859 (51%)	93 (49%)	122 (45%)	332 (47%)
Managed Care	1′453 (51%)	831 (49%)	95 (51%)	150 (55%)	377 (53%)
Number of Comorbidities	3 (1, 4)	3 (1, 4)	3 (2, 4)	3 (2, 5)	3 (1, 4)
*Death*					
In hospital	124 (4%)	80 (5%)	22 (3%)	3 (2%)	19 (7%)
In < 12 months	339 (12%)	180 (11%)	62 (8.7%)	24 (13%)	73 (27%)
In > 12 months	2′396 (84%)	1′430 (85%)	625 (88%)	161 (86%)	180 (66%)

^*a*^ Statistics presented: n (%); Median (IQR)

### Concentration effect

**Fig. 1:**
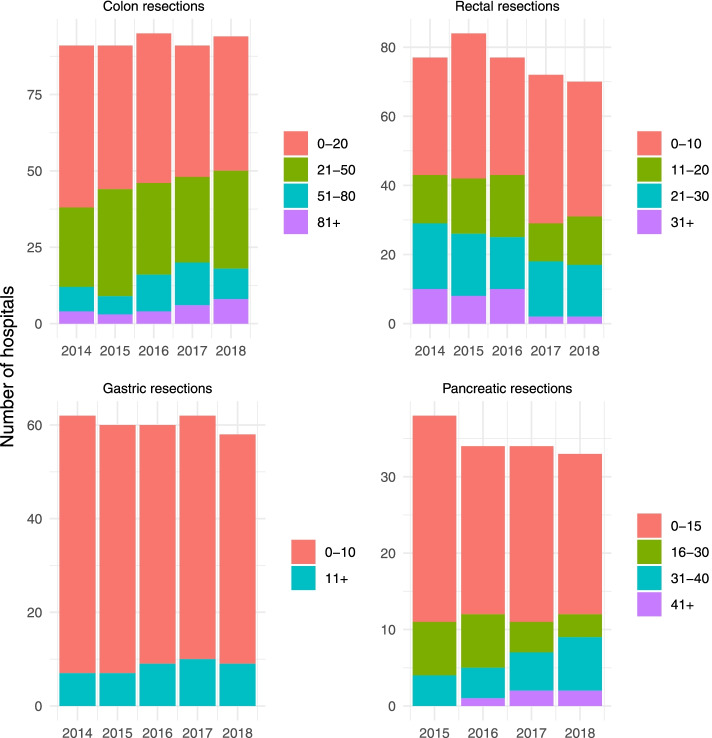
Changes in the number of abdominal surgery cases

**Fig. 2:**
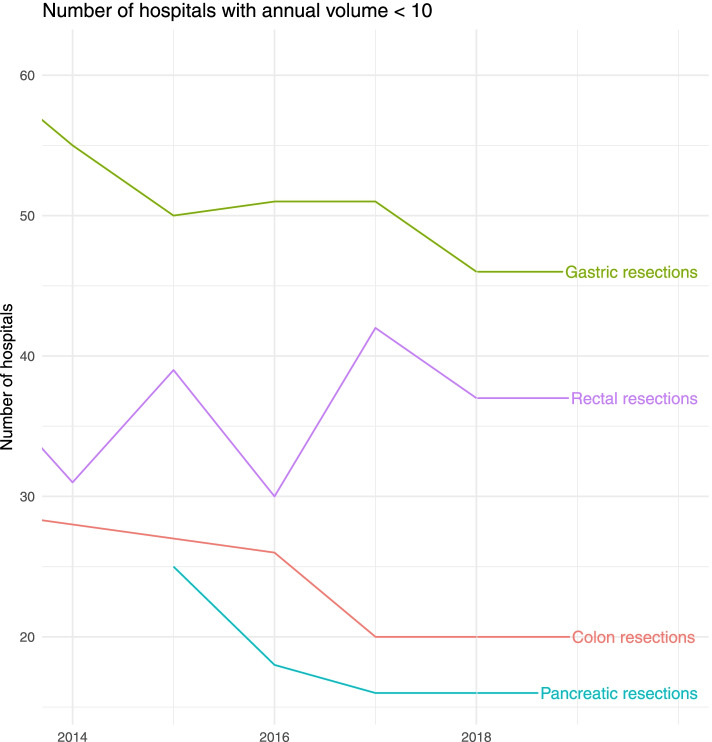
Changes in the number of hospitals with low hospital volumes

To examine a potential concentration effect, changes in the number of total resections per hospital and year were assessed, for each cancer type separately stratified by IQI specifications (Fig. 1). The mean number of resections per hospital increased from 26 (2014) to 30 (2018) for colon resections, from 19 (2014) to 12 (2018) for rectal resections, from 5 (2014) to 6 (2018) for gastric resections and from 12 (2015) to 16 (2018) for pancreatic resections. Simultaneously, a reduction of very low volume hospitals (< 10 resection per year) providing colon, gastric and pancreatic resections was observed (Fig. 2). Thus, treatment concentration was mainly observed for colon, gastric, and pancreatic resections. In contrast, changes in hospital volume were less noticeable for rectal resections.

To understand the hospital volume-outcome relationship, inpatient treatment outcomes as well as the outcomes in the twelve months after hospital discharge were differentiated according to hospital volumes and are shown in Tables 2 and 3. In general, the GEE models revealed no significant differences between the volume categories of the four cancer entities, with few exceptions in cost (Additional file 1: Tables A1-A4). Nevertheless, certain statistically insignificant trends and patterns in outcome parameters could be identified from the purely descriptive statistics (Tables 2 and 3).

**Table 2:** Volume – Inpatient outcomes

**Colon resections**						
**Characteristics**	**Overall** *N* = 1′690^*1*^	**≤20** *N* = 319^*1*^	**21–50** *N* = 626^*1*^	**51–80** *N* = 388^*1*^	**> 80** *N* = 357^*1*^	***p*-value**^**2**^ ***≤20*** 21–50 51–80 **>** 80
Inpatient cost	13′596 (10′397) / 10′402	13′215 (9′710) / 9′881	13′517 (9′490) / 11′311	14′056 (11′548) / 11′466	13′574 (11′188) / 9′215	0.053 **0.026** **0.022**
Length of hospital stay	17 (12) / 12	16 (11) / 11	17 (11) / 13	16 (13) / 12	18 (14) / 13	0.079 0.408 0.076
mortality	80 (5%)	18 (6%)	31 (5%)	18 (5%)	13 (4%)	0.513 0.469 0.059
**Rectal resections**						
**Characteristics**	**Overall** *N* = 709^*1*^	**≤10** *N* = 76^*1*^	**11–20** *N* = 123^*1*^	**21–30** *N* = 151^*1*^	**> 30** *N* = 359^*1*^	***p*-value**^**2**^ **≤*10*** 11–20 21–30 **>** 30
Inpatient cost	15′077 (11′264) / 12′637	16′112 (10′407) / 13′223	12′956 (4′542) / 12′373	15′465 (8′407) / 13′497	15′422 (13′770) / 11′592	0.073 0.445 0.507
Length of hospital stay	18 (13) / 14	19 (12) / 14	17 (11) / 12	18 (11) / 15	18 (15) / 13	0.092 0.950 0.475
Mortality	22 (3%)	4 (5%)	4 (3%)	6 (4%)	8 (2%)	0.856 0.746 0.433
**Gastric resections**						
**Characteristics**	**Overall** *N* = 188^*1*^	**≤10** *N* = 108^*1*^		**> 10** *N* = 80^*1*^		***p*-value**^**2**^ **≤*10*** **>** 10
Inpatient cost	18′883 (15′332) / 14′089	18′956 (16′884) / 14′145		18′784 (13′051) / 14′053		0.320
Length of hospital stay	20 (15) / 15	20 (17) / 14		19 (13) / 16		0.474
Mortality	3 (2%)	2 (2%)		1 (1%)		0.581
**Pancreatic resections**						
**Characteristics**	**Overall** *N* = 272^*1*^	**≤15** *N* = 82^*1*^	**16–30** *N* = 77^*1*^	**31–40** *N* = 88^*1*^	**> 40** *N* = 25^*1*^	***p*-value**^**2**^ **≤*15*** 16–30 31–40 > 40
Inpatient cost	22′084 (13′524) / 19′770	21′421 (12′131) / 20′739	21′907 (10′234) / 19′595	21′989 (11′981) / 20′542	25′143 (26′557) / 16′098	0.706 0.234 0.949
Length of hospital stay	22 (13) / 19	22 (13) / 19	24 (13) / 21	21 (11) / 18	21 (21) / 14	0.122 0.790 0.122
Mortality	19 (7%)	7 (9%)	7 (9%)	4 (5%)	1 (4%)	0.936 0.301 0.646

^*1*^ Indicated parameters: Mean (St.dev.) / median; n (%)

^*2*^ *P* values are based on the GEE models. Detailed results of the models are documented in the Additional file 1: Appendix. Reference categories are shown in italics

**Table 3:** Hospital Volume - Follow-Up cost

**Colon resections**						
**Characteristics**	**Overall** *N* = 1′690^*1*^	**≤20** *N* = 319^*1*^	**21–50** *N* = 626^*1*^	**51–80** *N* = 388^*1*^	**> 80** *N* = 357^*1*^	***p*-value**^**2**^ ***≤20*** 21–50 51–80 **>** 80
Outpatient cost^*3*^	18′189 (18′607) / 11′286	16′728 (16′670) / 10′615	18′972 (19′600) / 11′425	20′409 (20′546) / 12′692	15′793 (15′837) / 10′135	0.200 **0.018** 0.125
Medication cost^*3*^	6′685 (10′921) / 2′725	6′340 (9′598) / 2′679	7′304 (12′463) / 2′798	7′653 (11′773) / 2′887	4′930 (7′641) / 2′382	0.603 0.345 **0.046**
Inpatient cost^*3*^	7′121 (12′317) / 2′272	5′853 (9′481) / 982	7′067 (12′648) / 2′544	7′510 (12′252) / 2′754	7′882 (13′845) / 2′374	0.137/0.575 0.297/0.060 0.441/**0.007**
Mortality	260 (15%)	52 (16%)	106 (17%)	58 (15%)	44 (12%)	0.878 0.697 0.485
**Rectal resections**						
**Characteristics**	**Overall** *N* = 709^*1*^	**≤10** *N* = 76^*1*^	**11–20** *N* = 123^*1*^	**21–30** *N* = 151^*1*^	**> 30** *N* = 359^*1*^	***p*-value**^**2**^ **≤*10*** 11–20 21–30 **>** 30
Outpatient cost^*3*^	22′142 (18′675) / 15′927	22′502 (19′891) / 15′515	20′950 (17′444) / 16′209	25′249 (19′272) / 19′853	21′179 (18′516) / 14′927	0.488 0.300 0.477
Medication cost^*3*^	6′995 (10′918) / 2′862	6′195 (9′678) / 2′261	5′996 (9′048) / 3′060	8′107 (12′406) / 3′411	7′010 (11′037) / 2′780	0.900 0.689 0.931
Inpatient cost^*3*^	10′566 (13′681) / 6′732	12′827 (18′931) / 7′327	8′990 (9′400) / 6′043	11′940 (16′847) / 7′281	10′083 (12′101) / 6′618	0.059/0.666 0.159/0.082 0.130/0.052
Mortality	84 (12%)	15 (20%)	18 (15%)	17 (11%)	34 (10%)	0.666 0.082 0.051
**Gastric resections**						
**Characteristics**	**Overall** *N* = 188^*1*^	**≤10** *N* = 108^*1*^		**> 10** *N* = 80^*1*^		***p*-value**^**2**^ **≤*10*** **>** 10
Outpatient cost^*3*^	19′603 (16′368) / 15′887	18′584 (16′054) / 15′800		20′961 (16′798) / 16′133		0.642
Medication cost^*3*^	7′500 (9′679) / 3′552	7′066 (9′138) / 3′762		8′078 (10′397) / 3′417		0.465
Inpatient cost^*3*^	8′959 (24′482) / 2′880	11′213 (31′501) / 3′066		5′954 (8′139) / 2′661		0.923/0.310
Mortality	27 (14%)	16 (15%)		11 (14%)		0.888
**Pancreatic resections**						
**Characteristics**	**Overall** *N* = 272^*1*^	**≤15** *N* = 82^*1*^	**16–30** *N* = 77^*1*^	**31–40** *N* = 88^*1*^	**> 40** *N* = 25^*1*^	***p*-value**^**2**^ **≤*15*** 16–30 31–40 > 40
Outpatient cost^*3*^	29′313 (18′863) / 27′111	27′702 (22′681) / 20′451	28′291 (19′090) / 24′070	32′009 (16′007) / 30′133	28′508 (13′796) / 28′253	0.577 0.061 0.430
Medication cost^*3*^	11′924 (11′356) / 9′277	10′506 (12′245) / 7′033	12′204 (13′279) / 8′728	13′480 (9′503) / 10′930	10′450 (7′572) / 8′630	0.189 **0.010** 0.592
Inpatient cost^*3*^	8′486 (9′063) / 6′118	5′946 (6′676) / 3′886	9′739 (9′803) / 7′689	9′879 (10′047) / 8′167	8′147 (8′885) / 5′486	0.213/**0.034** 0.821/**0.001** 0.845/0.313
Mortality	92 (34%)	28 (34%)	26 (34%)	31 (35%)	7 (28%)	0.699 0.704 0.575

^*1*^ Indicated parameters: Mean (St.dev.) / median; n (%)

^*2*^ *P* values are based on the GEE models. *P*-values for inpatient follow-up cost are displayed for both, the binomial and gaussian models. Detailed results of the models are documented in the Appendix. Reference categories are shown in italics

^*3*^ Only complete observations over the entire period are displayed (e.g., no deaths)

### Hospital volume - mortality

Even though high hospital volumes seemed to be associated with lower in-hospital mortality (Table 2), the results lack statistical significance. However, the differences between hospitals with low and high volumes were most pronounced for rectal resections. For those interventions, the survival probability increased by 3% (from 95 to 98%).

Comparable trends were observed for mortality during the follow-up period (Table 3): Survival was also moderately increased for hospitals with high volumes for most of the resections (colon, rectal, gastric). In contrast to in-hospital survival, the difference here was more pronounced between the low and high hospital volume categories in all service areas. The largest discrepancy in survival rates was observed in rectal resections (80% vs. 90%).

### Hospital volume - length of stay

**Fig. 3:**
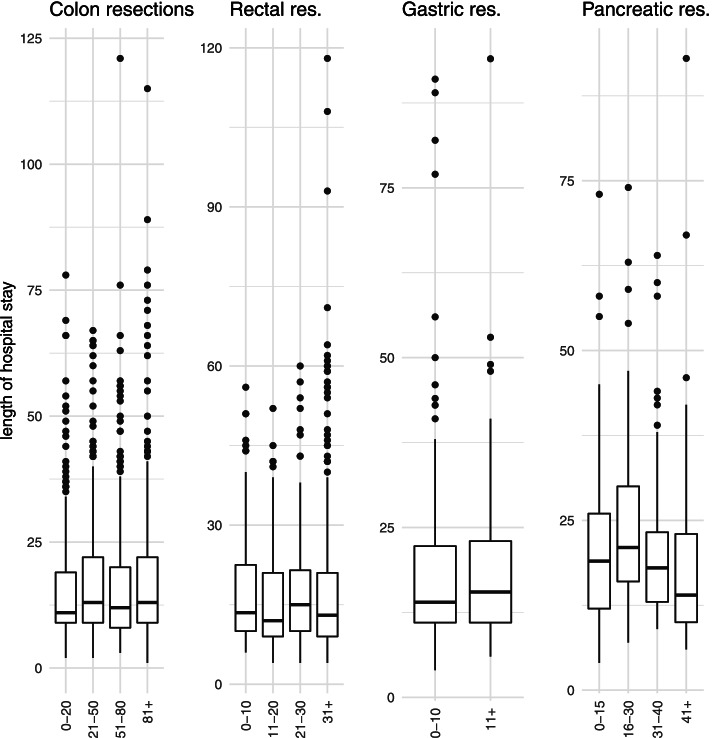
Hospital volume and length of hospital stay

Regarding length of hospital stay, the analysis did not show a consistent pattern with increasing hospital volume (Table 2, Fig. 3). Nevertheless, length of stay appeared to decrease with increasing hospital volume, predominantly for pancreatic resections. Similarly, rectal resections showed a comparable, but less clear pattern. In contrast, this development could not be observed for colon and gastric resections, as evidenced by rather unchanged distributions of length of hospital stays across the four volume categories.

### Hospital volume - cost

In terms of cost in the follow-up period, both hospitals with low and high volumes demonstrated lower cost as compared to intermediate volume categories. In fact, the cost of most resections increased sequentially with increasing hospital volume until the cost decreased again in the high hospital volume category (colon, rectal, pancreatic). However, statistical significance was only observed for individual cost groups of colon resections (inpatient cost of the intervention; follow-up: medication and outpatient cost) and pancreatic resections (follow-up: medication and inpatient cost).

The similar buckling pattern emerged for most outpatient and medication cost. Regarding inpatient cost during hospital stay, comparable cost development in dependence of hospital volume were only observed in colon resections: the median of inpatient cost was lower for low (CHF 9′881) as well as for high hospital volumes (CHF 9′215) compared to intermediate hospital volumes (CHF 11′311, 11′466). This commonly described pattern was also found in the inpatient cost during the follow-up period after colon resections: Again, the cost of the low (CHF 982) and high hospital volume (CHF 2′374) categories were lower than the cost of the intermediate categories (2′754, 2′544). For gastric resections, the bicategorical comparison indicates higher follow-up cost for hospitals with less than 10 annual interventions (CHF 3′066 vs. CHF 2′661).

## Discussion

This study gives further insight into the link between hospital volumes and outcomes based on real-life data of patients with four different types of highly incident abdominal cancer.

The first result is that during the observed time period, a concentration of service provision could be observed for colon, gastric, and pancreatic cancer resections. As second result, we found a less clear and statistically insignificant relationship between increasing hospital volume and better outcomes as opposed to most previous studies.

Our analysis showed no clear relationship between hospital volume and mortality. In general, the present analysis is based on low hospital volumes compared to other countries and very low mortality rates (Table 1). This probably hampered the detection of significant differences. For example, Nimptsch & Mansky 2017 showed a negative association between hospital volume and hospital mortality for resections of the colon in Germany [45]. This effect is shown with convincing significance only from about 90 cases per year compared with the reference category of about 20 cases. Hospital volumes in the present study are remarkably smaller, and hospitals with 90 cases per year are at the upper limit of the hospital volume distribution. Another study from England shows an effect in gastric cancer of ≥80 versus < 20 cases per year [11]. Our hospital volumes range up to a maximum of 33 cases within this service provision area, well below the hospital volumes in the Coupland et al. study.

The observed concentration of service provision is consistent with the existing literature in the Swiss context [7, 30, 44]. Hence, a Swiss study makes clear that the concentration of medical services is almost exclusively observed in services with defined minimum case numbers and in the areas of highly specialized medicine (HSM) [44]. HSM concerns the area of rare, complex and expensive interventions and therapies (e.g. severe burns in children) [46].

The cited study is in line with our findings, as resections of abdominal cancer are linked to minimum case numbers and have been part of HSM since 2016 [47].

Such concentration trends in hospital care are a double-edged sword. From an economic point of view, higher hospital volumes can be advantageous, e.g., due to purchasing advantages, increased capacity utilization, and lower average cost (economies of scale) [43]. On the contrary, a concentration of treatment cases at fewer sites might be detrimental to accessibility. Despite this potential negative effect, a comprehensive analysis commissioned by the FOPH rated hospital accessibility as “very good” in 2016 [44].

A beneficial experience effect on treatment outcomes is documented in a large body of the literature. For instance, Güller et al. showed a significant negative effect of hospital volume on in-hospital mortality rate after esophageal, gastric, rectal, and pancreatic resection [7]. The discrepancy between the Güller study and our results may be explained by a different observation period (1999–2012) as compared to our study (2014–2018), different patient identification methods (ICD-10) as compared to our study (IQI-specifications), and a more rigorous and comprehensive control for patient-sided variables that are likely to highly correlate with outcome in our study. Similarly, Krautz et al. showed a clear correlation between hospital volume and in-hospital mortality and postoperative complications for pancreatic surgery in Germany [40]. However, the relationship between incident abdominal cancer cases and the number of hospitals is markedly different in Germany as compared to Switzerland. Thus, the incident abdominal cancer cases are 150 times higher in the German study (5′403/year) as opposed to our study (45/year), whereas the average number of hospitals in one observation year is 5 times higher (178 vs. 35). Furthermore, a systematic review based in 19 studies conducted by Giwa et al. indicates reduced postoperative length of stay with increasing hospital volume [48]. However, the results are not directly comparable to our findings, as Giwa and colleagues included studies that were subject to different contextual conditions, such as a higher overall incidence of abdominal cancer resections and higher hospital volumes. There are two possible scientifically supported explanations for the gradual hospital volume-quality relationship: At the individual level, surgeons can reduce blood loss, operative time, and intraoperative and postoperative complications by gaining more experience in the field [20, 27, 49, 50]. From a structural perspective, hospitals with high volumes can contribute to better postoperative outcomes by providing expertise through multidisciplinary teams [12, 24, 28]. Studies have shown that collaboration between radiation oncologists, radiologists, medical oncologists, gastroenterologists, interventional radiologists, and pathologists is critical for the treatment of colon, rectal, gastric, and pancreatic cancers [2, 50].

Our finding that small hospitals can demonstrate comparable outcomes to larger hospitals is in line with individual studies. Thus, there is literature suggesting no significant differences in mortality between hospital volume categories in the first month after surgery [22, 23]. In line with our findings, further studies observe no relationship between hospital volume and postoperative morbidity [41–46], indirect proxy of corresponding cost [51–56]. To the best of our knowledge, there are no studies available so far that would allow an adequate comparison of the association between hospital volume and postoperative cost in the Swiss context. There are also research-based explanations for the finding that smaller hospitals can demonstrate similar results to larger hospital entities: several studies make clear that not necessarily only the volume per se, but rather the degree and extent of clinical infrastructure, resources and expertise of hospitals are decisive for treatment success [22, 23]. Thus, smaller regional hospitals with small volumes but excellent facilities and care processes can ensure equally good outcomes as hospitals with larger volumes. Our results imply that it is unlikely that patient outcomes will systematically improve if only unidimensional measures such as minimal hospital volumes are implemented, at least in healthcare settings with comparably low case numbers and a high dispersion of hospital infrastructure. Although this approach allows differentiating groups of hospitals with different levels of service provision, individual hospitals with low hospital volumes may also achieve outcomes above average outcomes. Therefore, switching from a categorization of hospitals based on hospital volumes to a categorization based on outcome quality could avoid this issue, which has already been shown to be successful in a Dutch study [57].

The results of this study should be interpreted in the context of a few important limitations. Firstly, the IQI system is not static and its specifications are constantly being further developed. Thus, in the given observation period, the versions for abdominal cancer have also changed, which might also contribute to temporal changes of hospital volumes to a small extent. Nevertheless, these diagnosis specifications provide an accurate categorization of the patient population and determination of hospital volumes with only little variation.

Secondly, compared to other countries, Switzerland faces generally low hospital volumes. This is particularly the case for gastric resections. Due to the relatively recent introduction of the DRG system and initial data inconsistencies, on which the given IQI specifications are based on, the observation period could, however, not be further extended. Thirdly, hospital volumes provided by the FOPH were aggregated at the hospital level, although some hospitals include multiple sites (e.g., Hirslanden Group, Insel Group). Rather, it is recommended to summarize hospital volumes at the hospital unit level to approximate the experience of a hospital unit more accurately. Fourthly, a limitation of the present investigation arises from information, which cannot be ascertained from the database, such as surgeon experience, presence of multidisciplinary teams, or existing infrastructure. It is therefore not possible to determine to what extent these factors may have influenced our analysis. To the best of our knowledge, however, the FOPH dataset is the most comprehensive, national dataset containing relevant information around hospital volumes in Switzerland, despite those minor shortcomings.

## Conclusion

In summary, the results indicate concentration trends of the hospital landscape, however, they lack evidence for a hospital volume-outcome relationship with respect to individual treatment outcomes. Thus, focusing on minimal case numbers as a single regulatory instrument for improving outcomes in cancer surgery is not efficient in healthcare settings with low absolute incident case numbers, high dispersion of hospital infrastructure and small-scale regional entities. Future studies should additionally take into account supply-related factors (i.e., the accessibility of hospitals, the availability of medical staff, medical equipment) to shed light on which further aspects affect treatment quality in hospitals, allowing for optimal hospital planning.

## Supplementary Information

**Additional file 1: Appendix Table A1.** Colon resections. **Table A2.** Rectal resections. **Table A3.** gastric resections. **Table A4.** Pancreatic resections.

## Data Availability

The data that support the findings of this study are available from Helsana (https://www.helsana.ch/en/helsana-group). Restrictions apply to the availability of these data, which were used under license for the current study and therefore are not publicly available. However, data are available from the authors upon reasonable request and with permission of Helsana (gesundheitskompetenz@helsana.ch).

## Abbreviations

HSM: Highly Specialized Medicine
IQI: Inpatient Quality Indicators
FOPH: Federal Office of Public Health
FOS: Federal Office of Statistics

## References

[1] Douek M, Taylor I (2003). Good practice and quality assurance in surgical oncology. Lancet Oncol.

[2] Hohenberger W, Merkel S, Hermanek P (2013). Volume and outcome in rectal cancer surgery: the importance of quality management. Int J Color Dis.

[3] Falzone L, Salomone S, Libra M (2018). Evolution of Cancer pharmacological treatments at the turn of the third millennium. Front Pharmacol.

[4] Wyld L, Audisio RA, Poston GJ (2015). The evolution of cancer surgery and future perspectives. Nat Rev Clin Oncol.

[5] Meagher AP (1999). Colorectal cancer: is the surgeon a prognostic factor? A systematic review. Med J Aust.

[6] Aquina CT, Kelly KN, Probst CP, Iannuzzi JC, Noyes K, Langstein HN (2015). Surgeon volume plays a significant role in outcomes and cost following open incisional hernia repair. J Gastrointest Surg.

[7] Güller U, Warschkow R, Ackermann CJ, Schmied B, Cerny T, Ess S (2017). Lower hospital volume is associated with higher mortality after oesophageal, gastric, pancreatic and rectal cancer resection. Swiss Med Wkly.

[8] Hanchanale VS, Javlé P (2010). Impact of hospital provider volume on outcome for radical urological cancer surgery in England. Urol Int.

[9] Yeo HL, Abelson JS, Mao J, O'Mahoney PRA, Milsom JW, Sedrakyan A (2017). Surgeon annual and cumulative volumes predict early postoperative outcomes after rectal Cancer resection. Ann Surg.

[10] Dikken JL, Dassen AE, Lemmens VEP, Putter H, Krijnen P, van der Geest L (2012). Effect of hospital volume on postoperative mortality and survival after oesophageal and gastric cancer surgery in the Netherlands between 1989 and 2009. Eur J Cancer.

[11] Brusselaers N, Mattsson F, Lagergren J (2014). Hospital and surgeon volume in relation to long-term survival after oesophagectomy: systematic review and meta-analysis. Gut..

[12] Markar SR, Karthikesalingam A, Thrumurthy S, Low DE (2012). Volume-outcome relationship in surgery for esophageal malignancy: systematic review and meta-analysis 2000-2011. J Gastrointest Surg.

[13] Coupland VH, Lagergren J, Lüchtenborg M, Jack RH, Allum W, Holmberg L (2013). Hospital volume, proportion resected and mortality from oesophageal and gastric cancer: a population-based study in England, 2004-2008. Gut..

[14] Hata T, Motoi F, Ishida M, Naitoh T, Katayose Y, Egawa S, Unno M (2016). Effect of hospital volume on surgical outcomes after Pancreaticoduodenectomy: a systematic review and Meta-analysis. Ann Surg.

[15] Yoshioka R, Yasunaga H, Hasegawa K, Horiguchi H, Fushimi K, Aoki T (2014). Impact of hospital volume on hospital mortality, length of stay and total costs after pancreaticoduodenectomy. Br J Surg.

[16] Birkmeyer JD, Stukel TA, Siewers AE, Goodney PP, Wennberg DE, Lucas FL (2003). Surgeon volume and operative mortality in the United States. N Engl J Med.

[17] Birkmeyer JD, Siewers AE, Finlayson EVA, Stukel TA, Lee LF, Ida B (2002). Hospital volume and surgical mortality in the United States. New England Journal of Medicine..

[18] Begg CB, Cramer LD, Hoskins WJ, Brennan MF (1998). Impact of hospital volume on operative mortality for major Cancer surgery. JAMA..

[19] Guller U, Safford S, Pietrobon R, Heberer M, Oertli D, Jain NB (2005). High hospital volume is associated with better outcomes for breast cancer surgery: analysis of 233,247 patients. World J Surg.

[20] Mamidanna R, Ni Z, Anderson O, Spiegelhalter D, Bottle A, Aylin P (2016). Surgeon volume and Cancer Esophagectomy, gastrectomy, and pancreatectomy: a population-based study in England. Ann Surg.

[21] Munasinghe A, Markar SR, Mamidanna R, Darzi AW, Faiz OD, Hanna GB (2015). Is it time to centralize high-risk Cancer Care in the United States? Comparison of outcomes of Esophagectomy between England and the United States. Ann Surg..

[22] Schell MT, Barcia A, Spitzer AL, Harris HW, Maddern GJ (2008). Pancreaticoduodenectomy: volume is not associated with outcome within an academic health care system. HPB Surg.

[23] Joseph B, Morton JM, Hernandez-Boussard T, Rubinfeld I, Faraj C, Velanovich V (2009). Relationship between hospital volume, system clinical resources, and mortality in pancreatic resection. J Am Coll Surg.

[24] Derogar M, Blomberg J, Sadr-Azodi O (2015). Hospital teaching status and volume related to mortality after pancreatic cancer surgery in a national cohort. Br J Surg.

[25] Sutton JM, Wilson GC, Wima K, Hoehn RS, Cutler Quillin R, Hanseman DJ (2015). Readmission after Pancreaticoduodenectomy: the influence of the volume effect beyond mortality. Ann Surg Oncol.

[26] Ess S, Joerger M, Frick H, Probst-Hensch N, Vlastos G, Rageth C (2011). Predictors of state-of-the-art management of early breast cancer in Switzerland. Ann Oncol.

[27] Derogar M, Sadr-Azodi O, Johar A, Lagergren P, Lagergren J (2013). Hospital and surgeon volume in relation to survival after esophageal cancer surgery in a population-based study. J Clin Oncol.

[28] Pecorelli N, Balzano G, Capretti G, Zerbi A, Di Carlo V, Braga M (2012). Effect of surgeon volume on outcome following pancreaticoduodenectomy in a high-volume hospital. J Gastrointest Surg.

[29] Liu C-J, Chou Y-J, Teng C-J, Lin C-C, Lee Y-T, Hu Y-W (2015). Association of surgeon volume and hospital volume with the outcome of patients receiving definitive surgery for colorectal cancer: a nationwide population-based study. Cancer..

[30] Bähler C, Blozik E, Hertle D, Jamieson A, Migliazza K, Näpflin M, Repschläger U, Schulte C, Wende D, Wilke F (2021). Stationäre Mindestmengen in Deutschland und der Schweiz: Zwischen Evidenz und Praxis.

[31] Zahnd D (2020). Mindestfallzahlen im Spital: Stand der Umsetzung in der Schweiz: Eine gesamtschweizerische Analyse betreffend die Umsetzung der GDK-Empfehlungen.

[32] Institut für Qualität und Wirtschaftlichkeit im Gesundheitswesen (IQWiG). Zusammenhang LM und Qualität bei komplexen Eingriffen am Pankreas - Rapid Report. 2021. http://www.iqwig.de/download/v19-03_zusammenhang-lm-und-qualitaet-bei-komplexen-eingriffen-am-pankreas_rapid-report_v1-1.pdf. Accessed 19 Nov 2021.

[33] Amiri M (2011). Stomach cancer mortality in the future: where are we going?. Int J Prev Med.

[34] Hashim D, Boffetta P, La Vecchia C, Rota M, Bertuccio P, Malvezzi M, Negri E (2016). The global decrease in cancer mortality: trends and disparities. Ann Oncol.

[35] Etemadi A, Safiri S, Sepanlou SG, Ikuta K, Bisignano C, Shakeri R (2020). The global, regional, and national burden of stomach cancer in 195 countries, 1990–2017: a systematic analysis for the global burden of disease study 2017. Lancet Gastroenterol Hepatol.

[36] Finks JF, Osborne NH, Birkmeyer JD (2011). Trends in hospital volume and operative mortality for high-risk surgery. N Engl J Med.

[37] Reames BN, Ghaferi AA, Birkmeyer JD, Dimick JB (2014). Hospital volume and operative mortality in the modern era. Ann Surg.

[38] Wasif N, Etzioni DA, Habermann E, Mathur A, Chang Y-H (2020). Correlation of Proposed Surgical Volume Standards for Complex Cancer Surgery with Hospital Mortality. J Am Coll Surg.

[39] Bundestamt für Gesundheit (2020). Qualitätsindikatoren der Schweizer Akutspitäler.

[40] Haller E, Watzke B, Blozik E, Rosemann T, Reich O, Huber CA, Wolf M (2019). Antidepressant prescription practice and related factors in Switzerland: a cross-sectional analysis of health claims data. BMC Psychiatry.

[41] Huber CA, Schwenkglenks M, Rapold R, Reich O (2014). Epidemiology and costs of diabetes mellitus in Switzerland: an analysis of health care claims data, 2006 and 2011. BMC Endocr Disord.

[42] Bundestamt für Gesundheit. Krankenversicherung. http://www.bag.admin.ch/bag/de/home/versicherungen/krankenversicherung.html. Accessed 16 Jun 2021.

[43] Martin Albrecht, Stefan Loos, Sebastian Irps, Jannis Bernhard. Qualitätsverbesserung durch Leistungskonzentration in der stationären Versorgung: Bestandsaufnahme, Erfolgsfaktoren und Hemmnisse. 2021. http://www.vdek.com/content/dam/vdeksite/vdek/verband/zukunftsforum/2021/IGES_Gutachten_2021.pdf.

[44] Gruber J.LS. Ergebnisbericht Sekundärdaten-Analysen zur Veränderung in der Spitallandschaft und zum Zugang zur stationären Versorgung. Studie innerhalb der Evaluation «Auswirkungen der KVG-Revision Spitalfinanzierung auf die Spitallandschaft und die Sicherstellung der stationären Versorgung. 2018. http://www.bag.admin.ch/dam/bag/de/dokumente/e-f/evalber-kuv/kvg-spitalf/2018-sekundaerdatenanalyse-ergebnisbericht.pdf.download.pdf/2018-ergebnisbericht-sekunddatenanalyse-d%20.pdf. Accessed 24 Nov 2021.

[45] Nimptsch U, Mansky T (2017). Hospital volume and mortality for 25 types of inpatient treatment in German hospitals: observational study using complete national data from 2009 to 2014. BMJ Open.

[46] Konferenz der kantonalen Gesundheitsdirektorinnen und -direktoren. Hochspezialisierte Medizin. http://www.gdk-cds.ch/de/hochspezialisierte-medizin. Accessed 23 Jun 2021.

[47] GDK Schweizerische Konferenz der kantonalen Gesundheitsdirektorinnen und -direktoren. Komplexe hochspezialisierte Viszeralchirurgie: Erläuternder Bericht für die Leistungszuteilung. 2019. http://www.gdk-cds.ch/de/hochspezialisierte-medizin/bereiche/komplexe-hochspezialisierte-viszeralchirurgie. Accessed 21 Nov 2021.

[48] Giwa F, Salami A, Abioye AI (2018). Hospital esophagectomy volume and postoperative length of stay: a systematic review and meta-analysis. Am J Surg.

[49] Aquina CT, Probst CP, Becerra AZ, Iannuzzi JC, Kelly KN, Hensley BJ (2016). High volume improves outcomes: the argument for centralization of rectal cancer surgery. Surgery..

[50] Hall GM, Shanmugan S, Bleier JIS, Jeganathan AN, Epstein AJ, Paulson EC (2016). Colorectal specialization and survival in colorectal cancer. Color Dis.

[51] Smith DL, Elting LS, Learn PA, Raut CP, Mansfield PF (2007). Factors influencing the volume-outcome relationship in gastrectomies: a population-based study. Ann Surg Oncol.

[52] Reid-Lombardo KM, Gay G, Patel-Parekh L, Ajani JA, Donohue JH, Gastric Patient Care Evaluation Group from the Commission on Cancer (2007). Treatment of gastric adenocarcinoma may differ among hospital types in the United States, a report from the National Cancer Data Base. J Gastrointest Surg.

[53] Ghaferi AA, Birkmeyer JD, Dimick JB (2011). Hospital volume and failure to rescue with high-risk surgery. Med Care.

[54] Reavis KM, Hinojosa MW, Smith BR, Wooldridge JB, Krishnan S, Nguyen NT (2009). Hospital volume is not a predictor of outcomes after gastrectomy for neoplasm. Am Surg.

[55] Murata A, Muramatsu K, Ichimiya Y, Kubo T, Fujino Y, Matsuda S (2015). Influence of hospital volume on outcomes of laparoscopic gastrectomy for gastric cancer in patients with comorbidity in Japan. Asian J Surg.

[56] Kuwabara K, Matsuda S, Fushimi K, Ishikawa KB, Horiguchi H, Fujimori K (2009). Hospital volume and quality of laparoscopic gastrectomy in Japan. Dig Surg.

[57] Wouters MWJM, Karim-Kos HE, Le Cessie S, Wijnhoven BPL, Stassen LPS, Steup WH (2009). Centralization of esophageal cancer surgery: does it improve clinical outcome?. Ann Surg Oncol.

[58] Federal Council. Federal act of 30 September 2011 on research involving human beings (human research act, HRA). 2019. from: http://www.admin.ch/opc/en/classified-compilation/20061313/index.html. Accessed 14 Oct 2021.

