# Supplementary material for: Does hospital volume affect outcomes after abdominal cancer surgery: an analysis of Swiss health insurance claims data
Source: BMC Health Serv Res. 2022 Feb 26;22:262. doi: 10.1186/s12913-022-07513-5 (PMC8881861; doi:10.1186/s12913-022-07513-5)
Supplement: Supplementary file 1 — Additional file 1: Appendix Table A1. Colon resections. Table A2. Rectal resections. Table A3. gastric resections. Table A4. Pancreatic resections. [file 12913_2022_7513_MOESM1_ESM.docx]

**Table A1 – Colon resections**

| Response variable | family | Hospital vol  21-50 | Hospital vol  51-80 | Hospital vol  >80 | age | gender | French | Italian | pccl | Number of comorbidities |
| --- | --- | --- | --- | --- | --- | --- | --- | --- | --- | --- |
| Hospital stay | | | | | | | | | | |
| Inpatient cost (box cox *λ* = -1.31) | gaussian | 0.00000019 (0.0000001);  p = *0.0525* | 0.00000024 (0.00000011);  p = **0.0261** | 0.00000028 (0.00000012);  p = **0.0222** | 0 (0);  p = 0.2139 | -0.00000004 (0.00000007);  p = 0.5161 | -0.00000005 (0.0000001);  p = 0.5972 | -0.00000051 (0.00000014);  p = **0.0003** | 0.00000072 (0.00000002);  p = **<0.0001** | 0.00000003 (0.00000002);  p = *0.0983* |
| Length of hospital stay (box cox  *λ* = -0.18) | gaussian | 0.0373 (0.0212); p = *0.079* | 0.0201 (0.0243); p = 0.4079 | 0.0464 (0.0261); p = *0.0757* | 0.005 (0.0007);  p = <0.0001 | 0.0112 (0.0158); p = 0.4802 | -0.0403 (0.0254);  p = 0.1128 | 0.0275 (0.0296); p = 0.3526 | 0.1175 (0.0046); p = **<0.0001** | 0.0043 (0.0039);  p = 0.2727 |
| mortality | binomial | 0.81 (0.44, 1.52),  p = 0.5135 | 0.77 (0.37, 1.58), p = 0.4688 | 0.43 (0.18, 1.03), p = *0.0587* | 1.05 (1.02, 1.08),  p = **0.0007** | 0.54 (0.32, 0.88), p = 0.0146 | 1.67 (0.82, 3.41),  p = 0.1575 | 2.29 (1.04, 5.03), p = **0.0391** | 41.48 (5.74, 299.83),  p = **0.0002** | 1.14 (1.02, 1.27),  p = **0.0177** |
| ****Follow Up (12 months)**** | | | | | | | | | | |
| Outpatient cost (box cox  *λ*) = 0.11) | gaussian | 0.245 (0.191); p = 0.1995 | 0.484 (0.204);  p = **0.0176** | -0.342 (0.222);  p = 0.1246 | -0.065 (0.006);  p = <0.0001 | -0.064 (0.131);  p = 0.6239 | 0.813 (0.179);  p = **<0.0001** | 0.68 (0.221);  p = **0.0021** | 0.603 (0.041);  p = **<0.0001** | 0.275 (0.031);  p = **<0.0001** |
| Medication cost (box cox  *λ* = 0.14) | gaussian | 0.16 (0.31);  p = 0.6032 | 0.32 (0.33);  p = 0.3451 | -0.72 (0.36);  p = **0.0463** | -0.09 (0.01); p = **<0.0001** | -0.43 (0.22);  p = *0.0507* | 0.64 (0.29);  p = **0.0274** | 0.59 (0.37);  p = 0.1121 | 0.85 (0.07);  p = **<0.0001** | 0.49 (0.05);  p = **<0.0001** |
| Inpatient cost | binomial | 1.27 (0.93, 1.74),  p = 0.1372 | 1.2 (0.85, 1.69), p = 0.2972 | 1.16 (0.8, 1.68), p = 0.4405 | 1.01 (1, 1.02),  p = **0.0081** | 1.32 (1.06, 1.65), p = **0.0134** | 0.85 (0.61, 1.17),  p = 0.3196 | 0.83 (0.56, 1.23), p = 0.3512 | 1.39 (1.3, 1.5),  p = **<0.0001** | 1.12 (1.06, 1.19),  p = **<0.0001** |
| Inpatient cost (box cox  *λ* = 0.11) | gaussian | 0.149 (0.266); p = 0.5753 | 0.5241 (0.279); p = *0.0603* | 0.8623 (0.3201); p = **0.0071** | -0.0077 (0.0084);  p = 0.359 | 0.0102 (0.1848); p = 0.956 | 0.0895 (0.291); p = 0.7583 | -0.3991 (0.3112);  p = 0.1997 | 0.4501 (0.0636); p = **<0.0001** | 0.0912 (0.0447);  p = 0.0413 |
| Mortality 12 m | binomial | 1.03 (0.7, 1.52),  p = 0.8777 | 0.92 (0.59, 1.42),  p = 0.6968 | 0.83 (0.5, 1.39), p = 0.485 | 1.05 (1.03, 1.07),  p = **<0.0001** | 0.73 (0.55, 0.98), p = **0.034** | 0.85 (0.54, 1.34),  p = 0.4904 | 0.91 (0.54, 1.53), p = 0.7307 | 2.83 (2.07, 3.87), p = **<0.0001** | 1.03 (0.96, 1.11),  p = 0.3468 |
| *General:*  GEE models with hospitals as cluster ID  Model specifications:  Dependent variables of the Gaussian Models are box-cox transformed  Binomial Models: odds ratio (confidence intervall)  Gaussian Models: coefficient (standard error)  Reference category hospital volume = 0 - 20  Reference category gender = male  Reference category language region = German  For follow up cost: only cases with complete observation period (i.e. no deaths) | | | | | | | | | | |

**Table A2- Rectal resections**

| Response variable | family | Hospital vol  11-20 | Hospital vol  21-30 | Hospital vol  >30 | age | gender | French | Italian | pccl | Number of comorbidities |
| --- | --- | --- | --- | --- | --- | --- | --- | --- | --- | --- |
| Hospital stay | | | | | | | | | |  |
| Inpatient cost (box cox  *λ* = -1.42) | gaussian | -0.0000001 (0.00000006);  p = *0.0725* | 0.00000004 (0.00000005);  p = 0.4454 | -0.00000003 (0.00000005);  p = 0.5069 | 0 (0);  p = 0.3994 | -0.00000003 (0.00000003);  p = 0.2991 | 0.00000008 (0.00000005);  p = *0.0886* | -0.00000015 (0.00000006);  p = **0.0095** | 0.00000015 (0.00000001);  p = **<0.0001** | 0.00000001 (0.00000001);  p = 0.1155 |
| Lengths of hospital stay (box cox  *λ* = -0.37) | gaussian | -0.0444 (0.0263); p = *0.0916* | -0.0015 (0.0242); p = 0.9504 | -0.0154 (0.0215); p = 0.4749 | 0.0004 (0.0006);  p = 0.4784 | -0.0077 (0.0137); p = 0.5739 | -0.027 (0.0221); p = 0.2217 | 0.0205 (0.024);  p = 0.3924 | 0.0668 (0.004);  p = **<0.0001** | 0.0106 (0.0035);  p = **0.0023** |
| mortality | binomial | 0.87 (0.2, 3.88), p = 0.8596 | 0.8 (0.2, 3.15),  p = 0.7457 | 0.61 (0.18, 2.08), p = 0.4332 | 1.15 (1.07, 1.23),  p = **<0.0001** | 0.2 (0.07, 0.59), p = **0.0035** | 1.52 (0.34, 6.77), p = 0.5862 | 0 (0, 0),  p = **<0.0001** | 12.8 (1.95, 84.01),  p = **0.0079** | 1.04 (0.81, 1.34),  p = 0.7473 |
| Follow Up (12 months) | | | | | | | | | |  |
| outpatient cost (box cox  *λ* = 0.14) | gaussian | -0.343 (0.494);  p = 0.488 | 0.47 (0.453);  p = 0.2996 | -0.305 (0.428);  p = 0.4765 | -0.05 (0.011); p = **<0.0001** | -0.548 (0.241);  p = **0.0232** | 0.912 (0.367);  p = **0.0131** | 0.253 (0.447);  p = 0.5717 | 0.642 (0.071);  p = **<0.0001** | 0.201 (0.069);  p = **0.0036** |
| Medication cost (box cox  *λ* = 0.11) | gaussian | 0.31 (0.5);  p = 0.5387 | 0.83 (0.49);  p = *0.0885* | 0.38 (0.46);  p = 0.413 | -0.06 (0.01); p = **<0.0001** | -0.73 (0.25);  p = **0.0031** | 0.63 (0.37);  p = *0.0941* | -0.08 (0.44);  p = 0.8616 | 0.54 (0.08);  p = **<0.0001** | 0.28 (0.07);  p = **<0.0001** |
| Inpatient cost | binomial | 0.44 (0.18, 1.03),  p = 0.0586 | 0.55 (0.24, 1.27),  p = 0.1592 | 0.55 (0.26, 1.19),  p = 0.1303 | 1.01 (0.99, 1.03),  p = 0.2184 | 0.58 (0.39, 0.87),  p = **0.0079** | 1.09 (0.58, 2.07),  p = 0.7896 | 1.33 (0.6, 2.97), p = 0.4869 | 1.17 (1.04, 1.31), p = **0.0104** | 1.03 (0.93, 1.15),  p = 0.5521 |
| Inpatient cost (box cox *λ* = 0.03) | gaussian | -0.02 (0.2);  p = 0.9004 | 0.08 (0.2);  p = 0.6887 | -0.02 (0.18);  p = 0.9312 | 0 (0);  p = 0.8584 | -0.17 (0.1);  p = *0.0988* | -0.16 (0.19);  p = 0.3869 | 0.03 (0.14);  p = 0.856 | 0.08 (0.03);  p = **0.0052** | 0.09 (0.03);  p = **0.0014** |
| Mortality 12 m | binomial | 0.84 (0.37, 1.89), p = 0.6661 | 0.5 (0.23, 1.09), p = *0.0823* | 0.49 (0.24, 1.01), p = *0.0516* | 1.06 (1.03, 1.09),  p = **<0.0001** | 0.79 (0.47, 1.34), p = 0.381 | 1.29 (0.57, 2.94), p = 0.5412 | 0.72 (0.24, 2.16), p = 0.5552 | 2.07 (1.48, 2.91), p = **<0.0001** | 0.93 (0.8, 1.08),  p = 0.3265 |
| *General:*  GEE models with hospitals as cluster ID  Model specifications:  Dependent variables of the Gaussian Models are box-cox transformed  Binomial Models: odds ratio (confidence interval)  Gaussian Models: coefficient (standard error)  Reference category hospital volume = 0 - 10  Reference category gender = male  Reference category language region = German  For follow up cost: only cases with complete observation period (i.e. no deaths) | | | | | | | | | | |

**Table A3- gastric resections**

| Response variable | family | Hospital vol  <10 | age | gender | French | Italian | pccl | Number of comorbidities |
| --- | --- | --- | --- | --- | --- | --- | --- | --- |
| Hospital stay | | | | | | | | |
| Inpatient cost (box cox  *λ* = -1.54) | gaussian | 0.00000001 (0.00000001);  p = 0.3202 | 0 (0);  p = 0.9529 | 0.00000001 (0.00000001);  p = 0.5563 | 0.00000001 (0.00000003);  p = 0.6895 | -0.00000005 (0.00000002);  p = **0.0257** | **0.00000003 (0);**  **p = <0.0001** | -0.00000001 (0); p = 0.1998 |
| Lengths of hospital stay (box cox  *λ* = -0.29) | gaussian | 0.022 (0.03);  p = 0.4735 | 0.001 (0.001);  p = 0.3312 | 0.047 (0.032);  p = 0.1429 | 0.038 (0.06);  p = 0.5216 | 0.064 (0.044);  p = 0.1466 | 0.083 (0.009);  p = **<0.0001** | 0.005 (0.009);  p = 0.5826 |
| mortality | binomial | 0.408 (0.017, 9.836),  p = 0.5808 | 1.086 (0.97, 1.216),  p = 0.1501 | 11.877 (0.512, 275.696),  p = 0.123 | 2.13 (0.142, 32.006),  p = 0.5844 | 11.567 (1.537, 87.021),  p = **0.0174** | >1000 (>1000, >1000), p = **<0.0001** | 1.818 (0.91, 3.629),  p = *0.0903* |
| Follow Up (12 months) | | | | | | | | |
| outpatient cost (box cox  *λ* = 0.18) | gaussian | 2.66 (5.7);  p = 0.6415 | -0.09 (0.16); p = 0.5828 | -0.69 (2.04);  p = 0.7355 | -1.3 (8.12);  p = 0.873 | 0.63 (6.46);  p = 0.922 | 0.57 (0.52);  p = 0.2694 | 0.31 (0.74);  p = 0.6789 |
| Medication cost (box cox  *λ* = 0.18) | gaussian | 0.75 (1.02);  p = 0.4649 | -0.04 (0.04); p = 0.3433 | -1.18 (0.95);  p = 0.2126 | -0.96 (1.2);  p = 0.4229 | -2.38 (1.75);  p = 0.1749 | 0.75 (0.25);  p = **0.003** | 0.48 (0.23);  p = **0.0347** |
| Inpatient cost | binomial | 0.97 (0.49, 1.92),  p = 0.923 | 1.02 (0.99, 1.05), p = 0.1442 | 1.76 (0.89, 3.46),  p = 0.1048 | 0.61 (0.22, 1.71),  p = 0.3456 | 0.47 (0.18, 1.21),  p = 0.1166 | 1.25 (1.03, 1.52),  p = **0.0231** | 1.11 (0.95, 1.31),  p = 0.1969 |
| Inpatient cost (box cox  *λ* = -0.09) | gaussian | -0.098 (0.097);  p = 0.3096 | -0.001 (0.003);  p = 0.8488 | -0.031 (0.091); p = 0.7356 | -0.015 (0.141);  p = 0.9167 | -0.122 (0.187); p = 0.5144 | 0.043 (0.026);  p = *0.0994* | 0.018 (0.023);  p = 0.4321 |
| Mortality 12 m | binomial | 0.936 (0.371, 2.359),  p = 0.8883 | 0.995 (0.959, 1.032),  p = 0.7797 | 1.231 (0.5, 3.029),  p = 0.6508 | 0.915 (0.245, 3.416),  p = 0.8953 | 1.198 (0.327, 4.396), p = 0.785 | 2.472 (1.504, 4.063), p = **0.0004** | 1.114 (0.906, 1.369),  p = 0.3068 |
| *General*:  GEE models with hospitals as cluster ID  Model specifications:  Dependent variables of the Gaussian Models are box-cox transformed  Binomial Models: odds ratio (confidence interval)  Gaussian Models: coefficient (standard error)  Reference category hospital volume = 0 - 10  Reference category gender = male  Reference category language region = German  For follow up cost: only cases with complete observation period (i.e. no deaths) | | | | | | | | |

**Table A4 – Pancreatic resections**

| Response variable | family | Hospital vol  16-30 | Hospital vol  31-40 | Hospital vol  >40 | age | gender | French | Italian | pccl | Number of comorbidities |
| --- | --- | --- | --- | --- | --- | --- | --- | --- | --- | --- |
| Hospital stay | | | | | | | | | | |
| Inpatient cost (box cox \(\lambda\) = -0.84) | gaussian | 0.0000047 (0.0000125);  p = 0.7063 | 0.000015 (0.0000127);  p = 0.2343 | 0.0000013 (0.0000204);  p = 0.949 | 0.000001 (0.0000005);  p = *0.0594* | 0.0000089 (0.0000103);  p = 0.386 | 0.000001 (0.0000228);  p = 0.9645 | -0.0000164 (0.0000157);  p = 0.2964 | 0.0000454 (0.0000067);  p = <0.0001 | -0.0000008 (0.0000027);  p = 0.7596 |
| Lengths of hospital stay (box cox *λ* = -0.13) | gaussian | 0.0809 (0.0524);  p = 0.1224 | -0.0134 (0.0503);  p = 0.7895 | -0.1458 (0.0941);  p = 0.1215 | 0.0022 (0.0021); p = 0.302 | 0.0793 (0.0408); p = 0.0519 | 0.1708 (0.0841); p = 0.0422 | 0.0191 (0.0761); p = 0.8018 | 0.1189 (0.0205); p = <0.0001 | 0.0072 (0.0099);  p = 0.471 |
| mortality | binomial | 0.96 (0.33, 2.79),  p = 0.9359 | 0.51 (0.14, 1.83), p = 0.3008 | 0.6 (0.07, 5.34), p = 0.6462 | 1.03 (0.97, 1.09), p = 0.3028 | 0.88 (0.36, 2.14), p = 0.774 | 0 (0, 0),  p = <0.0001 | 1.71 (0.43, 6.77), p = 0.4415 | 7.28 (1.3, 40.73), p = 0.0239 | 0.97 (0.73, 1.28),  p = 0.8171 |
| Follow Up (12 months) | | | | | | | | | | |
| outpatient cost (box cox  *λ* = 0.34) | gaussian | 2.43 (4.37);  p = 0.5771 | 8.14 (4.33);  p = *0.0605* | 4.73 (5.99);  p = 0.4296 | -0.39 (0.16);  p = **0.0154** | 1.46 (3.14);  p = 0.6417 | -2.55 (8.11);  p = 0.753 | -4.8 (4.84);  p = 0.3214 | 2.65 (1.43);  p = *0.064* | 1.88 (0.79);  p = **0.0168** |
| Medication cost (box cox  *λ* = 0.22) | gaussian | 1.84 (1.4);  p = 0.1887 | 3.5 (1.36);  p = **0.0099** | 1.1 (2.05);  p = 0.5916 | -0.08 (0.06);  p = 0.1743 | 0.46 (0.95);  p = 0.627 | 1.44 (2.33);  p = 0.5379 | -0.66 (1.68);  p = 0.6939 | 1.16 (0.44);  p = **0.0091** | 0.54 (0.25);  p = **0.031** |
| Inpatient cost | binomial | 1.98 (0.68, 5.8), p = 0.2131 | 0.9 (0.37, 2.18), p = 0.8219 | 0.88 (0.23, 3.28),  p = 0.8454 | 1.01 (0.97, 1.04), p = 0.763 | 0.94 (0.44, 2), p = 0.8671 | 0.29 (0.1, 0.91), p = **0.0338** | 0.23 (0.08, 0.65),  p = **0.0059** | 1.02 (0.72, 1.44),  p = 0.9299 | 1.11 (0.94, 1.32),  p = 0.2248 |
| Inpatient cost (box cox  *λ* = 0.11) | gaussian | 0.9 (0.4);  p = **0.0344** | 1.5 (0.4);  p = **0.0005** | 0.7 (0.6);  p = 0.3126 | 0 (0); p = 0.1573 | 0.1 (0.3);  p = 0.7499 | -0.2 (0.6);  p = 0.6956 | -0.5 (0.6);  p = 0.4415 | 0.1 (0.1);  p = 0.5688 | 0.2 (0.1);  p = **0.0036** |
| Mortality 12 m | binomial | 0.88 (0.45, 1.71), p = 0.699 | 1.13 (0.59, 2.18), p = 0.7038 | 0.76 (0.29, 1.97), p = 0.575 | 1.04 (1.01, 1.07), p = **0.0054** | 1.43 (0.85, 2.41), p = 0.1815 | 0.92 (0.37, 2.27), p = 0.8533 | 1.27 (0.54, 2.98), p = 0.5768 | 1.44 (1.03, 2.02), p = **0.0315** | 0.97 (0.84, 1.11),  p = 0.6446 |
| *General*:  GEE models with hospitals as cluster ID  Model specifications:  Dependent variables of the Gaussian Models are box-cox transformed  Binomial Models: odds ratio (confidence interval)  Gaussian Models: coefficient (standard error)  Reference category hospital volume = 0 - 15  Reference category gender = male  Reference category language region = German  For follow up cost: only cases with complete observation period (i.e. no deaths) | | | | | | | | | | |
